# Case report: The first description of a Dieulafoy's lesion in the gastric mucosa of a dog

**DOI:** 10.3389/fvets.2022.932435

**Published:** 2022-08-22

**Authors:** Daniel Felipe Barrantes Murillo, Michael Tillson, Jennifer W. Koehler, Maninder Sandey

**Affiliations:** ^1^Department of Pathobiology, College of Veterinary Medicine, Auburn University, Auburn, AL, United States; ^2^Department of Clinical Sciences, College of Veterinary Medicine, Auburn University, Auburn, AL, United States

**Keywords:** Dieulafoy's lesion, stomach, domestic animal, dog, animal

## Abstract

An approximately 12-year-old, 31 kg, male neutered Labrador Retriever was presented to the referring hospital with an acute onset (less than 1 day) of hematemesis and melena. The dog was treated supportively for a presumptive gastric ulcer for 4 days with intravenous fluids, gastro protectants, such as pantoprazole, misoprostol, sucralfate, and barium, as well as an anti-emetic (maropitant) and analgesics (fentanyl, gabapentin, and tramadol). Throughout medical management, the dog continued to require blood transfusions approximately every 24 h. Given the poor medical response, the patient was subjected to an exploratory laparotomy. During surgery, a grossly raised, blister-like lesion on the mucosal surface of the stomach was appreciated on the lesser curvature of the stomach. A partial gastrectomy was performed, and the segment was submitted for histological evaluation. Histologically, there were multiple, tortuous, medium-caliber muscular arteries (>1.0 mm in diameter) in the submucosa. A single large-caliber artery (>0.75 mm in diameter) containing a partially occlusive thrombus extruded through the mucosa and projected on the ulcerated surface. The patient's signs were similar clinically and histopathologically to Dieulafoy's lesion in people. A Dieulafoy's lesion is a potentially life-threatening disorder that causes gastrointestinal (GI) hemorrhage. This lesion is characterized by a dilated, large-caliber, aberrant submucosal artery that erodes through the epithelium and ruptures, resulting in massive and potentially fatal hemorrhage. This lesion has never been documented previously in a dog.

## Introduction

Gastrointestinal (GI) hemorrhage is a significant cause of morbidity and mortality in dogs. It can be caused by various etiologies, such as coagulation disorders, neoplasia, toxin exposure, non-steroidal anti-inflammatory drug (NSAID) administration, hepatic or renal disease, hypoadrenocorticism, and vascular anomalies (1). Vascular anomalies are rarely reported as a cause of GI hemorrhage in dogs, with only a few case reports describing vascular ectasias in the colon (2, 3), cecum (4), and small intestine, such as jejunum (5, 6), and are summarized in Table 1. Vascular anomalies are more frequently diagnosed as a cause of GI bleeding in humans. Vascular anomalies described in people include varices, hemorrhoids, vascular ectasias, angiodysplasias, and Dieulafoy's lesions (7). Dieulafoy's lesion is diagnosed in approximately 1–6% of cases of acute GI bleeding in people (8, 9) and is characterized by a large, aberrant, tortuous artery in the stomach (most commonly) or other parts of the GI tract. To our knowledge, a lesion similar to Dieulafoy's lesion in people has never been reported in a dog. This case report describes the clinical, surgical, and histological features of a Dieulafoy's-like lesion in a geriatric canine patient.

**Table 1:** Vascular anomalies associated with gastrointestinal (GI) hemorrhage in dogs.

**Vascular anomalies in the gastrointestinal tract in dogs**
**Breed**	**Age**	**Sex**	**Anomaly**	**Reference**
Mixed-breed	8 years	Male	Colonic vascular ectasia (angiodysplasia)	(2)
English Springer Spaniel	8 months	Female	Colonic vascular ectasia (angiodysplasia)	(3)
Golden Retriever	8 years	Male	Cecal and colonic vascular ectasia	(4)
-	Juvenile	-	Jejunal arteriovenous fistula	(5)
Mixed-breed	7 months	Female	Small intestinal vascular ectasia	(6)

## Case presentation

An approximately 12-year-old, 31 kg, male neutered Labrador Retriever was presented to the referring hospital with an acute onset (less than 1 day) of hematemesis and melena. The patient had a history of carprofen, a non-steroidal anti-inflammatory (carprofen 4.4 mg/kg PO q24 h), and opioid (tramadol 2 mg/kg PO q12 h) administration intermittently over the past 2 years for arthritis. Both drugs were given at appropriate doses and frequencies and were given as needed, which was typically 3–5 times a month. The last dose of these drugs was given 5 days before the onset of clinical signs. Baseline bloodwork the morning after admission included a complete blood count (CBC), biochemistry panel, and coagulation panel (Table 2). CBC findings were consistent with regenerative anemia. The coagulation panel revealed increased fibrinogen and serum biochemistry profile with panhypoproteinemia, decreased calcium, cholesterol, and increased creatine kinase (CK). The overall results of baseline bloodwork were consistent with blood loss, with no signs of underlying organ dysfunction. Baseline cortisol was within the reference interval (78 mmol/L; reference interval 10–160), making hypoadrenocorticism unlikely. Thoracic and abdominal radiographs and abdominal ultrasound were within normal limits, except for the presence of gastric ileus. There was no overt evidence of masses suggesting neoplasia.

**Table 2:** Baseline blood work.

**Complete blood count (CBC)**
**Test**	**Result**	**Units**	**Reference interval**
RBC	**3.81**	× 10∧6/μL	6.02–8.64
HGB	**8.5**	g/dL	13.1–20.1
HCT	**27.0**	%	38.7–59.2
MCV	70.7	fL	60.5–73.8
MCH	22.2	pg	20.4–25.7
MCHC	**31.4**	g/dL	32.0–37.2
RDW	**19.2**	%	11.2–14.4
Platelet count	502	x 10∧3/μL	152–518
MPV	12.6	fL	8.0–14.6
RETIC_PCT	**4.25**		0.00–1.50
RETIC_ABS	**161.9**	x 10∧3/μL	0.0–60.0
WBC	**56.15**	x 10∧3/μL	5.09–17.41
**Coagulation profile**			
Platelet count	219	x 10∧3/μL	152–518
MPV	8.6	fL	8.0–14.6
PT TIME	7.6	sec	7.4–9.1
APTT	13.7	sec	11.6–14.0
Fibrinogen	213	mg/dL	101–156
Antithrombin	147	%	>150
D-Dimer	<250	ng/mL	0–250
PLASMA FDP	<5	μg/mL	0–5
**Serum chemistry profile**			
Total protein	**4.19**	g/dL	5.50–7.70
Albumin	**1.5**	g/dL	3.0–4.3
Globulin	2.7	g/dL	2.0–4.3
ALB/GLOB Ratio	**0.6**		0.7–1.9
ALK_PHOS	118.8	U/L	14–152
ALT	48	U/L	13–151
AST	**88**	U/L	18–55
Total bilirubin	0.12	mg/dL	0.00–0.20
CK	**1,713**	U/L	53–337
BUN	**6.1**	mg/dL	9.0–34.0
Creatinin	1.1	mg/dL	0.5–1.6
Calcium	**8.8**	mg/dL	9.6–12.0
Phosphorus	5.6	mg/dL	2.6–7.9
Glucose	109	mg/dL	76–116
CHOL	**110**	mg/dL	132–335
Bicarbonate	15.3	mmol/L	14.0–29.0
Sodium	148	mmol/L	142–151
Potassium	4.7	mmol/L	3.6–4.9
Chloride	115	mmol/L	105–117
Anion GAP	22.4		14.0–24.0
OSMOL (CALC)	292	mOsm/kg	284–314
S.IRON	**38**	μg/dL	76–229
LIPEMIA_INDEX	5		0–35
HEMOLYSIS_INDEX	23		0–56
ICTERUS_INDEX	0		0–0

Without an obvious underlying cause for the clinical signs, the dog was treated supportively for a presumptive gastric ulcer, with top differential etiologies being neoplasia (such as a gastrinoma) or chronic NSAID use. It was treated medically for 4 days with intravenous fluids, gastro protectants, such as pantoprazole, misoprostol, sucralfate, and barium, as well as an anti-emetic (maropitant) and analgesics (fentanyl, gabapentin, and tramadol). Throughout medical management, the dog continued to require blood transfusions approximately every 24 h based on the acute onset of pale mucous membranes, tachycardia, tachypnea, hypotension, hyperlactatemia, weakness, and a decreased packed cell volume (PCV) and total solids (TS). The dog received a total of 47 ml/kg of blood products (packed red blood cells and fresh whole blood) during this time. Between transfusions, the dog clinically looked well and was eating voluntarily but continued to have melena intermittently.

**Figure 1:**
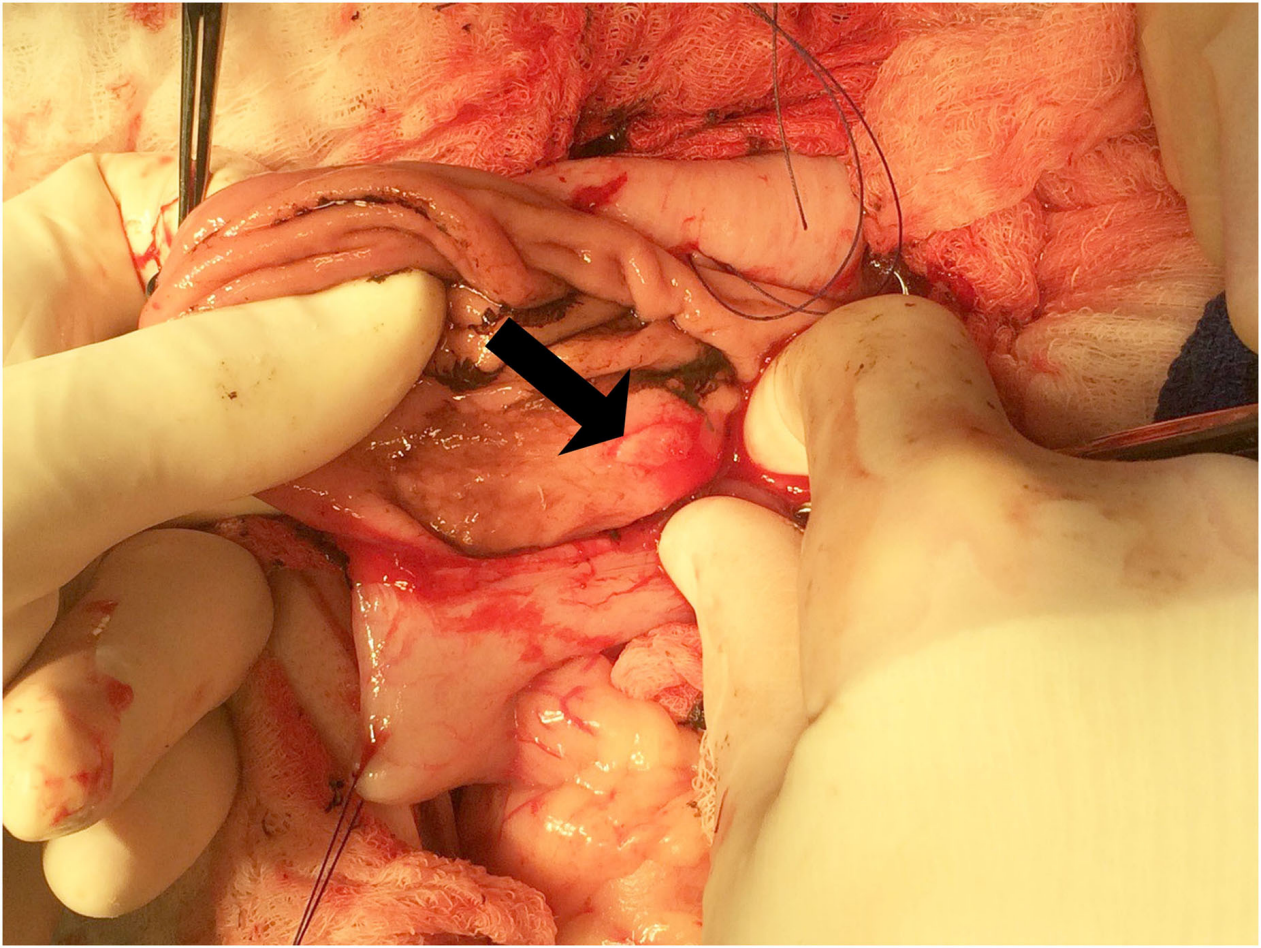
Exploratory laparotomy and gastrectomy. The grossly raised, blister-like lesion on the mucosal surface of the stomach can be seen on the lesser curvature of the stomach (arrow).

On day 4 of hospitalization, an upper gastrointestinal endoscopy was performed based on the poor response to medical management and continued need for blood transfusions. The gastric mucosa could not be adequately examined due to the presence of large blood clots in the stomach; thus, abdominal exploratory surgery was performed. There was approximately 500 ml of dark red, clotted blood within the gastric lumen. A small, 2 cm in diameter, round, light pink blister-like lesion was present on the mucosal surface of the stomach near the pyloric antrum, closely associated with the lesser curvature (Figure 1). The surrounding mucosa appeared normal. A small incision into the lesion revealed an artery that began to bleed profusely. A partial-thickness gastrectomy was performed to control hemorrhage and remove the lesion, which was submitted for histopathology. There were no other remarkable findings in the abdomen during exploratory surgery. The dog recovered uneventfully and did not require any additional blood transfusions during its hospital stay. Haematologic parameters and melena improved; however, the dog succumbed to complications associated with a surgical infection 12 days after surgery.

**Figure 2:**
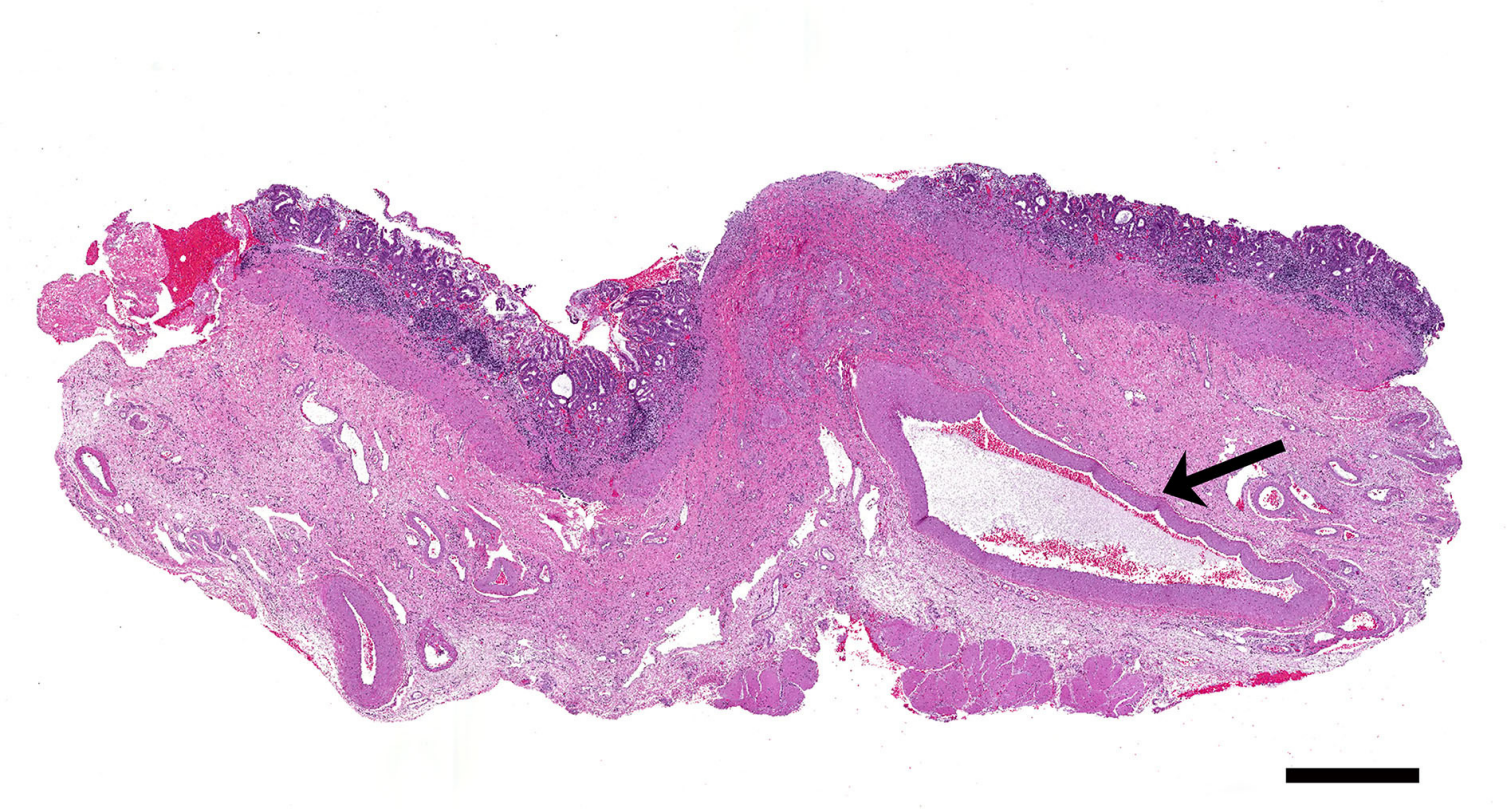
A large caliber artery (arrow) is present within the submucosa of the stomach. H&E. Bar = 1,000 μm.

**Figure 3:**
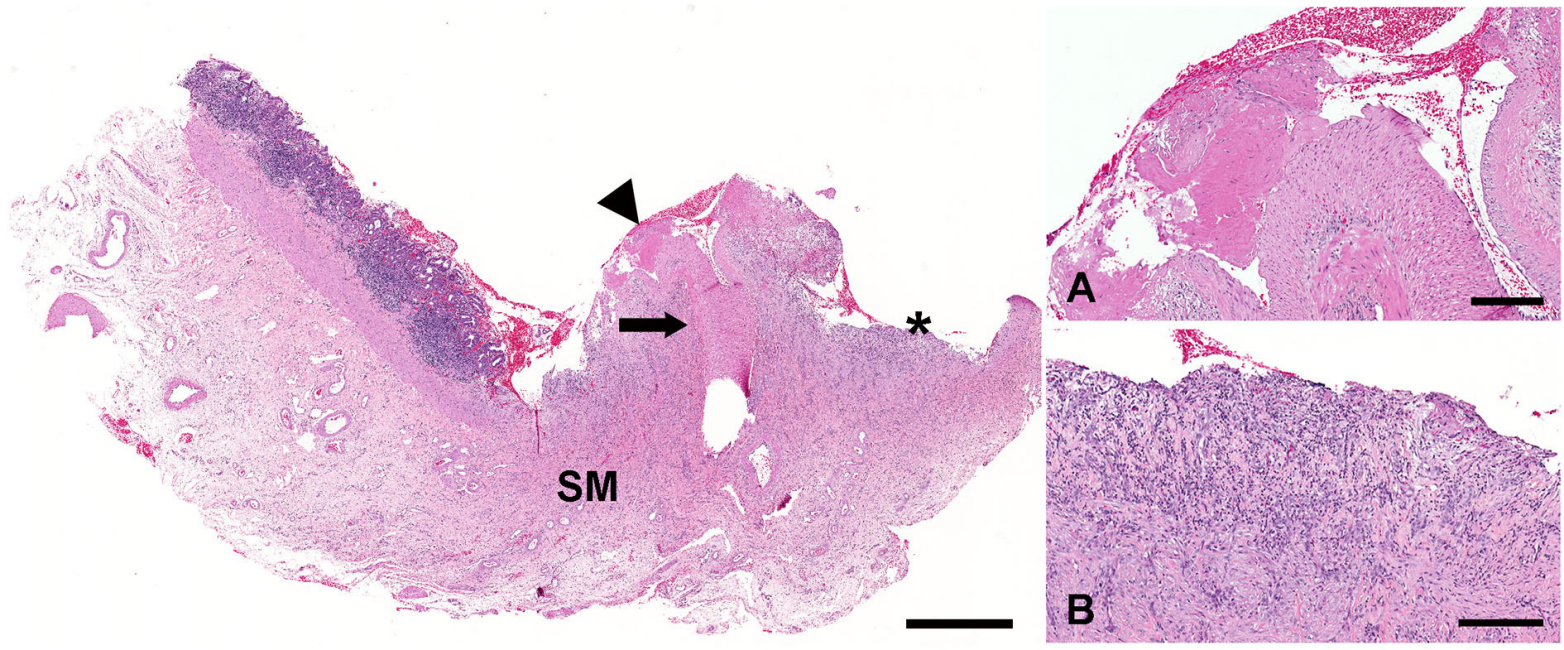
The large-caliber artery (arrow) located in the submucosa of the stomach (SM) erodes through the mucosa (arrowhead). The overlying gastric mucosa is ulcerated (asterisk). H&E. Bar = 1,000 μm. An aberrant artery has evidence of rupture and intravascular thrombosis **(A)**. H&E. Bar = 200 μm. The ulcer bed is composed of granulation tissue that is infiltrated by large numbers of lymphocytes, plasma cells, and neutrophils **(B)**. H&E. Bar = 200 μm.

Histologically, there were multiple cross and longitudinal sections of medium-caliber, tortuous muscular arteries (>1.0 mm in diameter) located in the submucosa subjacent to the grossly visible blister-like lesion (Figure 2). A single large-caliber artery (>0.75 mm in diameter) containing a partially occlusive thrombus extruded through the muscularis mucosae and projected through the mucosal ulcer (Figure 3). There was a focally extensive mucosal defect overlying the ectopic artery (Figure 3). The superficial lamina propria and submucosa were infiltrated by moderate to large numbers of lymphocytes and fewer macrophages, which multifocally also formed small lymphoid aggregates. However, the inflammation was restricted to the lamina propria and submucosa surrounding the ulcerated area.

## Discussion

This report describes an unusual case of life-threatening gastrointestinal hemorrhage in a dog caused by a rupture of an ectopic artery within the gastric submucosa. To our knowledge, there are no previous case reports describing an ectopic gastric artery leading to a potentially fatal hemorrhage in a dog. The patient's signs were similar clinically and histopathologically to Dieulafoy's lesion in people, which has also never been documented in a dog. A Dieulafoy's lesion is a potentially life-threatening disorder that accounts for 1–6% of all causes of gastrointestinal hemorrhage in humans (8, 9). This lesion is characterized by a dilated, large-caliber, aberrant submucosal artery that erodes through the epithelium and ruptures, resulting in massive and potentially fatal hemorrhage into the gastric lumen. The lesion is often (65% of cases) located in the proximal lesser curvature of the stomach (8), which is similar to the location of the lesion in our patient. Although most cases of Dieulafoy's lesion are reported in the stomach, it can occur in other parts of the gastrointestinal tract. A Dieulafoy's lesion has been described in the esophagus, small intestine, colon, or rectum (10). Men are affected approximately two times as often as women, and the lesion can occur at any age (10–12).

The cause of Dieulafoy's lesion is unknown, and various studies in people have failed to show an association with NSAID administration, chronic alcohol consumption, or other underlying diseases, such as hypertension, cardiac disorders, and renal failure. The vascular architecture of the lesser curvature of the stomach may explain its predisposition to develop this lesion. The most widely accepted theory is that Deulafoy's lesion is a congenital anomaly. When an aberrant submucosal artery traverses close enough to the mucosal surface, it becomes vulnerable to rupture. It is also believed that mucosal ulceration and ischemia may be the initial steps in the rupture of this submucosal blood vessel (13). The pulsatility of the aberrant submucosal arteries creates pressure on the overlying mucosa leading to hypoxia, contributing to the thinning of the mucosal wall and exposing the artery to the lumen contents (11). An exposed artery is vulnerable to erosion by the mechanical trauma of a food bolus (stomach) or stool (colon and rectum) (11). Vascular ectasia, ischemic injury-related cardiovascular disease, mucosal atrophy, and neoplasia are more common in aging patients and may be responsible for the increased incidence of this lesion in geriatric men (11, 14, 15). Comorbidities predisposing to bleeding have been identified in patients affected with Dieulafoy's lesion and include alcohol consumption, drug abuse, advanced liver disease, autoimmune disorders, cardiopulmonary conditions, diabetes mellitus, hyperlipidemia, concomitant peptic ulcer disease, and renal disease (11). Human patients with Dieulafoy's lesion display clinical signs of melena, hematochezia, anemia, and/or hematemesis and may also have weakness, fatigue, dizziness, or dyspnea (8).

Antemortem diagnosis of Dieulafoy's lesion is often made *via* upper GI endoscopy in 70% of the cases. However, lesions can be missed if they are small, not actively bleeding, or obscured by large amounts of blood in the lumen (10, 11). The use of endoscopy as a diagnostic tool has dramatically decreased the reported mortality from 80 to 8.3% reported in a study (12). There are three diagnostic criteria used for the diagnosis of Dieulafoy's lesion in people using endoscopy: (1) active arterial spurting or micropulsatile streaming from a mucosal defect <3 mm, or through normal surrounding mucosa, (2) visualization of a protruding vessel with or without bleeding, within a minute mucosal defect or through normal surrounding mucosa, and (3) the appearance of a fresh, densely adherent clot with a narrow point of attachment to a minute mucosal defect or normal mucosa (11, 15, 16). The dog described here met all three diagnostic criteria, although they were visualized either during surgery or with histopathology rather than endoscopy. Histopathologically, human cases of Dieulafoy's lesion have tortuous vessels within the submucosa of the stomach, which may protrude into the muscularis mucosa and may contain recent or recanalized thrombi. There is often no inflammation within the mucosal layer, an important feature that differentiates Dieulafoy's lesion from gastric ulcers (17). Additional histopathologic features that should not be present in cases of Dieulafoy's lesion are deep ulcerations, direct arterial penetration of the muscularis propria, vasculitis, aneurysm formation, or arteriosclerosis (18). Histologically, the current case meets the diagnostic criteria described for Dieulafoy's lesion. Current treatment options for Dieulafoy's lesion include partial gastrectomy and endoscopic mechanical methods, such as hemoclip application or band ligation (11).

## Conclusion

In conclusion, this case report describes a ruptured aberrant gastric artery, consistent with Dieulafoy's lesion in people, resulting in a massive and life-threatening gastric hemorrhage in a dog. Although rare, this type of vascular anomaly should be included in the differential diagnosis list in dogs that have experienced intermittent, profuse GI hemorrhage that is unresponsive to traditional medical management. Upper GI endoscopy may fail to identify the lesion in cases with marked intraluminal blood accumulation; thus, when evaluating gastric biopsy, pathologists should consider Dieulafoy's lesion as a possible differential when a large caliber-persistent artery traverse through the submucosa and mucosa.

## Data availability statement

The original contributions presented in the study are included in the article/supplementary material, further inquiries can be directed to the corresponding author.

## Ethics statement

Ethical review and approval were not required for this case because the dog was submitted for routine diagnostic post-mortem examination to the Department of Pathobiology and, as such, is not subject to animal ethics guidelines. The owners permitted the post-mortem evaluation.

## Author contributions

MT followed the clinical case. MS and JK performed the antemortem examination and final report. DB and MS prepared the manuscript and literature review. MS contributed to the design, supervised the study, and critically revised and edited the manuscript. DB, MT, JK, and MS reviewed the final submission. All authors read and approved the final manuscript.

## Conflict of interest

The authors declare that the research was conducted in the absence of any commercial or financial relationships that could be construed as a potential conflict of interest.

## Publisher's note

All claims expressed in this article are solely those of the authors and do not necessarily represent those of their affiliated organizations, or those of the publisher, the editors and the reviewers. Any product that may be evaluated in this article, or claim that may be made by its manufacturer, is not guaranteed or endorsed by the publisher.
